# Anti-Microbial and Remineralizing Properties of Self-Adhesive Orthodontic Resin Containing Mesoporous Bioactive Glass

**DOI:** 10.3390/ma14133550

**Published:** 2021-06-25

**Authors:** Aerin Choi, Kyung-Hyeon Yoo, Seog-Young Yoon, Bong-Soo Park, In-Ryoung Kim, Yong-Il Kim

**Affiliations:** 1 Department of Orthodontics, Dental Research Institute, Pusan National University, Yangsan 50612, Korea; pnudh_aerin@naver.com; 2 School of Materials Science and Engineering, Pusan National University, Busan 46241, Korea; seweet07@pusan.ac.kr (K.-H.Y.); syy3@pusan.ac.kr (S.-Y.Y.); 3 Department of Oral Anatomy, School of Dentistry, Pusan National University, Yangsan 50612, Korea; parkbs@pusan.ac.kr; 4 Dental and Life Science Institute, School of Dentistry, Pusan National University, Yangsan 50612, Korea

**Keywords:** self-adhesive resin, mesoporous bioactive glass nanoparticles, antibacterial, remineralization

## Abstract

Self-adhesive resins (SARs) contain adhesives, which simplify the procedures of resin application, and primers, which provide sufficient bonding ability. In this study, mesoporous bioactive glass nanoparticles (MBN) were added to a SAR to easily improve the physical properties and remineralization ability. The experimental resins comprised 1%, 3%, and 5% MBN mixed in Ortho Connect Flow (GC Corp, Tokyo, Japan). As the MBN content in the SAR increased, the microhardness increased, and a statistically significant difference was observed between the cases of 1% and 5% MBN addition. Shear bond strength increased for 1% and 3% MBN samples and decreased for 5% MBN. The addition of MBN indicated a statistically significant antibacterial effect on both gram-negative and gram-positive bacteria. The anti-demineralization experiment showed that the remineralization length increased with the MBN content of the sample. Through the above results, we found that SAR containing MBN has antibacterial and remineralization effects. Thus, by adding MBN to the SAR, we investigated the possibility of orthodontic resin development, wherein the strength is enhanced and the drawbacks of the conventional SAR addressed.

## 1. Introduction

Most recent research and innovative studies on dental materials have focused on simplifying the bonding procedure and improving bond durability to achieve rapid application in intraoral conditions [1]. Self-adhesive resins (SARs) are desirable materials for simplifying the treatment procedure because they do not require additional restorative adhesives to adhere to the dental substrate. Owing to the shear bond strength and marginal sealing potential of self-adhesive restorative materials, SARs are good candidate materials for clinical applications [2,3,4]. The chemical corrosion affected both polymeric matrix and filler particles. Salivary enzymes can soften the surface of dimethacrylate polymers by inducing hydrolysis of methacrylate ester bonds. Water sorption leads mainly to hydrolytic corrosion of silane coupling and reinforcing fillers [5]. Methacrylate monomers, such as bisphenol A-glycidyl methacrylate (Bis-GMA), may be released after polymerization. Bis-GMA functions to limit photopolymerization-induced volumetric shrinkage and to enhance resin reactivity. In order to avoid even the slightest influence of this corrosion, it should be avoided in a moist environment in the oral cavity [6]. Transbond XT (3M Unitek, Monrovia, CA, USA), which does not contain adhesive is used as a conventional restorative material in orthodontics. The evolution of dental materials used in clinical orthodontics has led to the production of lots of self-adhesive restorations, such as GC Ortho connect, Vertise^TM^ Flow (Kerr, Orange, CA, USA), Fusio Liquid Dentin (Pentron, Orange, CA, USA), Fuji II LC (GC Crop, Tokyo, Japan), and Activa (Pulpdent, Watertown, MA, USA). SARs provide adequate adhesion to mineralized dental structures by shortening the bonding time in clinics, where moisture control and isolation are difficult. Therefore, SARs have a significant advantage over conventional bonding materials [7]. However, SARs also have a disadvantage in that they adversely shorten the lifespan of the bonding materials and increase the microleakage level with a high internal stress, which may cause deformation due to wear, fatigue, thermal shrinkage, and expansion over time [8]. Similar to the mechanism of self-etching adhesives, SAR eliminates the risk of collagen network collapse caused by excessive drying after acid etching and rinsing; hence, SARs are designed to adhere to dentin and are not effective for enamel adhesion [9].

Mesoporous bioactive glass nanoparticles (MBNs) are bioactive substances consisting of SiO_2_, CaO, Na_2_O, and P_2_O_5_. MBNs have high chemical stability, mechanical stability, and effective bioactive functions, and they are widely used as biological materials [10]. They have the ability to remineralize enamel and dentin with high bioactivity, lower cytotoxicity to dental pulp stem cells, and antibacterial activity against intraoral bacteria [11,12]. Moreover, resin-modified glass ionomers and adhesives containing MBN are known to release calcium and phosphate, which improve the mechanical properties of demineralized hard tissues [11,12,13].

In orthodontic treatment, the most extensively used resin adhesives for bracket adhesion are composed of dimethacrylate monomers, which are hardened after polymerization and may involve adjacent enamel damage when the brackets detach after orthodontic treatment [14]. In contrast, self-adhesive materials can fail to maintain attachments in the long term owing to the aforementioned shortcomings of increased microleaks over time. However, this could also be an advantage given the nature of orthodontic bracket adhesion, as they would be easy to remove after the completion of orthodontic treatment.

Therefore, there is a need to develop self-adhesive materials for functional orthodontic treatment. By adding bioactive glass (BAG) to the well-known composition of existing SARs, a practitioner can perform the convenient operation of applying the SAR with additional antibacterial and remineralization abilities.

Innovative SARs for functional dental orthodontics may help minimize side effects, such as white spot lesions and caries, which inevitably occur during orthodontic treatment. Consequently, this study aimed to explore the possibility of developing orthodontic adhesives with remineralization abilities and antimicrobial capabilities, while enhancing the mechanical properties by adding MBN to Ortho Connect Flow (GC Crop, Tokyo, Japan). The specific objectives are: (1) to compare the mechanical and physical properties with those of existing corrective adhesive by varying the amount of MBN added to the self-adhesive materials; and (2) to test the prevention of demineralization, the capability of remineralization, and antibacterial ability of the orthodontic adhesive with MBN compared to the conventional orthodontic adhesive resin.

## 2. Materials and Methods

### 2.1. Mesoporous Bioactive Glass Nanoparticle Synthesis

MBNs were synthesized using a modified sol–gel method [15]. We added 20 mL ethanol (Samchun, Pyeongtaek, Korea), 2 mL aqueous ammonia (Samchun, Pyeongtaek, Korea), 10 mL 2-ethoxyethanol (Sigma-Aldrich, St. Louis, MO, USA), 2.54 g calcium nitrate tetrahydrate (Sigma-Aldrich, St. Louis, MO, USA), and 1 g hexadecyltrimethylammonium bromide (CTAB, Sigma-Aldrich, St. Louis, MO, USA) to 150 mL distilled water and stirred for 30 min at 600 rpm. Then, 5 mL of tetraethyl orthosilicate (TEOS; Sigma-Aldrich, St. Louis, MO, USA) was added and stirred for 30 min. Subsequently, 0.47 mL of triethyl phosphate (TEP; Sigma-Aldrich, St. Louis, MO, USA) was added and stirred at room temperature (around 20–22 °C) for 4 h. After the white precipitate was formed, it was washed in distilled water and dried for 24 h in an oven at 60 °C. Subsequently, heat treatment at 600 °C was performed for 5 h in a furnace [16]. Synthesized MBNs were analyzed by attenuated total reflectance Fourier-transform infrared spectroscopy (ATR-FTIR; Nicolet iS50, Thermo Fisher Scientific Co., Madison, WI, USA) using a built-in all-reflective diamond ATR module. The FTIR spectrum was recorded at 4000–400 cm^−1^ and 32 scans per second with a resolution of 4 cm^−1^. To observe the crystalline state, X-ray diffraction (XRD; Ultima IV, Rigaku, Tokyo, Japan) was conducted with Cu K_α_ radiation (λ = 0.154 nm). The diffractometer operated at 40 kV and 40 mA, discs were scanned at a rate of 4°/min. The particle size and morphology of MBN were examined using field emission transmission electron microscope (FE-TEM, JEM-2100F, JEOL, Tokyo, Japan) operated at 200 kV.

### 2.2. Preparation of Experimental Self-Adhesive Orthodontic Bonding Resin

To create experimental orthodontic bonding resin samples, the synthesized MBN was mixed with the SAR (Ortho Connect Flow; GC Corp, Tokyo, Japan, Table 1) in experimental ratios of 0 wt%, 1 wt%, 3 wt%, and 5 wt%, respectively. To prevent polymerization by light during mixing, SAR and MBN were added to a 2 mL black e-tube and mixed twice, for 10 s in each round of mixing, using a mixer (mixing speed; 2850 rotations per minute, 3M ESPE, Seefeld, Germany). These experimental orthodontic bonding resin used to bond metal brackets and make discs. After that light (1000 mW/cm^2^, VALO; Ultradent, South Jordan, UT, USA) was cured for 20 s on each tooth and discs. The characterization of the fabricated discs fabricated discs (10 mm) was analyzed by XRD and FTIR. The MBN placement in the resin structure was evaluated using the field emission scanning electron microscope (FE-SEM, S-4300, HITACHI, Tokyo, Japan) and micro-computed tomography (InspXio SMX-90CT, Shimadzu, Kyoto, Japan) at 90 kV, 109 μA condition.

**Figure 1:**
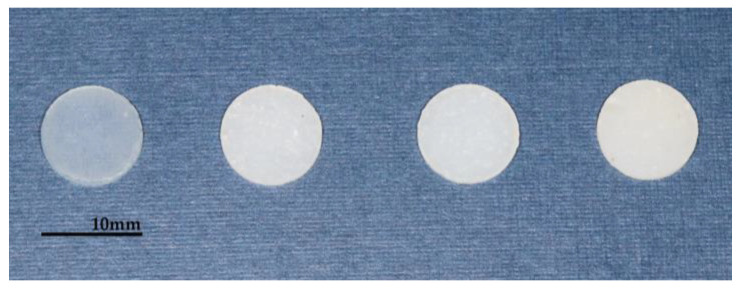
Resin Discs (SAR, SAR + 1% MBN, SAR + 3% MBN, SAR + 5% MBN, scale bar: 10 mm).

To compare the mechanical and biological properties of the experimental groups, two different sizes of resin discs were fabricated (5 mm and 10 mm diameters and 1 mm height, Figure 1). 

### 2.3. Microhardness

To measure the mechanical hardness of each sample group, a microhardness tester (MVK-H1, Mitutoyo, Kanagawa, Japan) was used to perform Vickers’ test for 20 discs (10 mm) per group. Microhardness was defined as the load on the surface area of the indented area. A load of 100 gf was used.

### 2.4. Degree of Conversion (DC)

The degree of conversion was calculated by the result of FTIR. This method utilizes the peak ratios of double bonds in aliphatic compounds (1640 cm^−1^) and aromatic compounds (1610 cm^−1^) to determine the DC. To determine the ratio of formed double bonds after polymerization per group, the absorbance spectra were measured for the methacrylate carbon double bond and internal standard prior to polymerization and after polymerization [17]. The DC was calculated using the equation shown below. Three data trials were conducted for each group (10 mm disc).

(1) $$\%DC=\left(1-\frac{\left(aliphatic\;C=C/aromatic\;C=C\right)\;polymer}{\left(aliphatic\;C=C/aromatic\;C=C\right)\;monomer}\right)\times 100$$

### 2.5. Anti-Bacterial Test

*Streptococcus mutans* (ATCC 25175; American Type Culture Collection (ATCC), Manassas, VA 201808, USA) and *Porphyromonas gingivalis* (KCTC 5352, Korean Collection for Type Cultures, Jeollabuk-do, Korea) were used for the antibacterial tests. *S. mutans* which is a major cariogen through its production of lactic acid was evaluated for the antibacterial ability of reducing WSL (white spot lesion) [18]. The *S. mutans*, the major etiological agent of WSL on the bacterial field, was used for the antibacterial test. *P. gingivalis* is the keystone pathogen of periodontitis, a chronic inflammatory disease that causes tooth loss and deterioration of gingiva [19]. Bacterial suspensions in sterile saline solution were prepared to an optical density equal to 0.5 McFarland standard. *S. mutans* was cultured in brain heart infusion at 37 °C and stored in an aerobic incubator. *P. gingivalis* was cultured in tryptic soy agar hemin menadione medium and stored at 37 °C in an anaerobic incubator. To examine the antimicrobial activity of the experimental orthodontic bonding resin, four different groups (control (SAR), SAR + 1% MBN, SAR + 3% MBN, SAR + 5% MBN) were used. Each disc (5 mm) was ethylene oxide (EO) gas sterilized and placed in a 96-well plate, and 1.0 × 10^5^ CFU/mL of *S. mutans* and *P. gingivalis* were added to each well. *S. mutans* and *P. gingivalis* were then cultured in an aerobic and anaerobic incubator at 37 °C, respectively. Each of the four experimental groups was incubated for 24 h, 48 h, and 72 h, and the absorbance at 405 nm was measured using a microplate reader (SpectraMax, iD3, BioTek, Winooski, VT, USA).

### 2.6. Shear Bond Strength (SBS) Test

Twenty premolars per group for orthodontic treatment were used in this experiment. To bond brackets to the teeth, 35% phosphoric acid gel (Ultra Etch; South Jordan, UT, USA) was applied for acid etching of the teeth for 30 s and then removed, rinsed, and dried. After confirming the chalky surface of the tooth after etching, without applying orthodontic adhesive, four groups of experimental orthodontic bonding resins were added to the bracket base corresponding to the long axis of the teeth. The access resins were removed, and each resin application was light-cured for 20 s. Samples were stored in distilled water for 24 h, and the shear bond strength (SBS) was measured using a universal testing machine (Instron, Canton, MA, USA). The SBS (MPa) was calculated by measuring the maximum load (N) with the crosshead at a speed of 1 mm/min divided by the bracket base surface area. The remaining resins were then evaluated using the adhesive remnant index (ARI) score, as shown in Table 2 [20].

### 2.7. Anti-Demineralization Test

The pH cycling method was used to test the anti-demineralization and remineralization effects of the experimental orthodontic bonding resins [21]. After the bracket was bonded, the samples were stored in distilled water for 24 h, followed by a cycle of submerging samples in demineralization solution (Biosesang, Seongnam, Korea) for 6 h, and 18 h in remineralization solution (Biosesang, Seongnam, Korea), which was repeated for 14 days. Between the solution changes, the samples were transferred to distilled water for 1 min, washed, and dried. The solutions were replaced every seven days. After pH cycling, samples were scanned using micro-computed tomography (micro CT; InspXio SMX-90CT, Shimadzu, Kyoto, Japan) at 90 kV, 109 μA condition. Micro CT data were analyzed using ImageJ software (National Institutes of Health, Bethesda, MD, USA). Various sizes of scanned CTs were corrected using a scale bar. To account for sound enamel in the data, a brightness of 87% was defined as sound enamel, and data exceeding the criteria were considered to have shown enamel loss. The remineralization length of each sample was measured, which was defined as the end point of orthodontic bonding resins to the accounted sound enamel. 

### 2.8. Statistical Analysis

After mechanical and biological tests, a one-way analysis of variance (ANOVA) and Tukey’s post hoc comparison were performed. The ARI scores between groups were compared using the Kruskal–Wallis and Mann–Whitney tests. A *p*-value of less than 0.05 was statistically significant. All statistical analyses were performed using SPSS version 21.0 (IBM, Armonk, NY, USA).

## 3. Results

### 3.1. Characterization of MBNs and Fabricated Resin Discs

**Figure 2:**
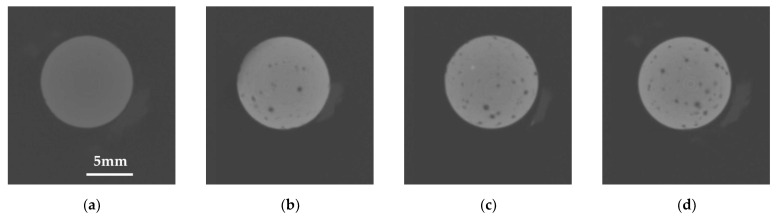
Micro CT images of the resin discs; (**a**) control (SAR); (**b**) SAR + 1% MBN; (**c**) SAR + 3% MBN; and (**d**) SAR + 5% MBN.

**Figure 3:**
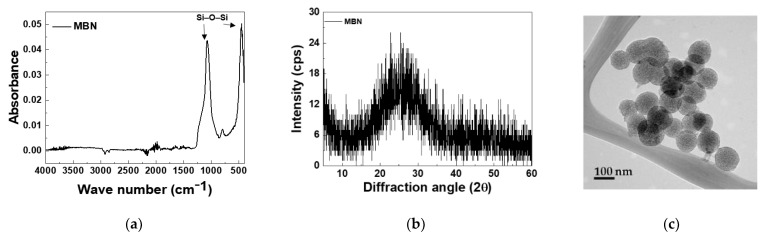
(**a**) FT-IR spectra; (**b**) XRD pattern; and (**c**) TEM image of the synthesized MBN.

The results of the characterization of synthesized MBN are shown in Figure 2 and Figure 3. The micro-CT images of the resin discs are shown in Figure 2. To identify the chemical structures of MBNs, FT-IR spectra are shown in Figure 3a. The Si–O–Si rocking vibration was detected at 472 cm^−1^. The band at 1078 cm^−1^ was assigned to the Si–O–Si asymmetrical stretching vibrations [22,23]. In addition, in the XRD result (Figure 3b), an amorphous phase peak was detected in the 2θ range of 15° to 40°. The morphology of MBNs is shown in Figure 3c. Synthesized MBNs had spherical morphology and a mesopore structure. Additionally, the diameter of the particle was around 100 nm. 

**Figure 4:**
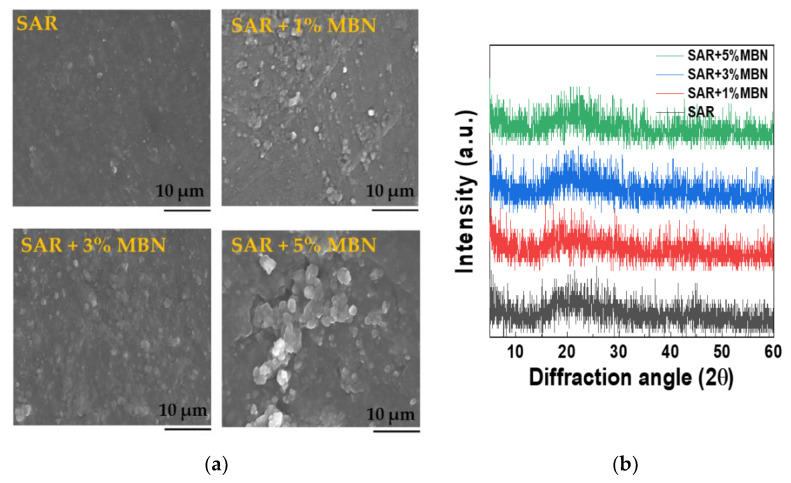
(**a**) SEM images; and (**b**) XRD patterns of SAR with different amounts of MBN.

Figure 4 shows the surface images of SAR and MBN contained in the SAR resin discs. For the SAR + 5%MBN disc, MBN particles agglomerated in the resin structure. In the XRD patterns, all samples with different amounts of MBNs revealed an amorphous phase.

### 3.2. Shear Bond Strength (SBS)

**Figure 5:**
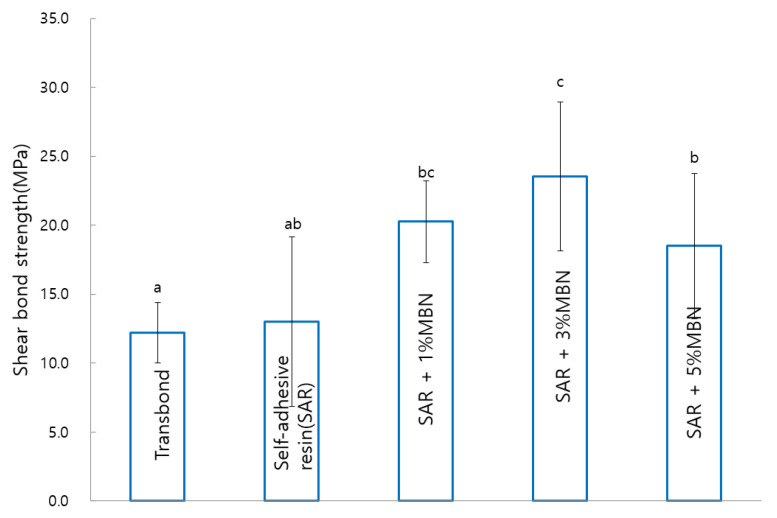
Comparison of shear bond strength (SBS) between the commercial control (Transbond XT), control (SAR), and MBN-treated SAR groups. Labels with the same letters indicate no statistically significant differences between groups. One-way ANOVA was performed (*n* = 20).

There were significant sheer bond length differences between the untreated control (Transbond XT, 12.21 ± 2.21 MPa; SAR, 13.01 ± 5.97 MPa) and MBN-treated groups of SAR + 1% MBN (20.25 ± 2.95 MPa), SAR + 3% MBN (23.55 ± 5.39 MPa), and SAR + 5% MBN (18.49 ± 5.26 MPa) (*p* < 0.05). As the amount of MBN increased from 1% to 3%, the SBS correspondingly increased; however, the 5% MBN group showed a slight decrease in the shear bond strength (Figure 5). 

### 3.3. ARI Score

There was no significant difference in the groups with respect to the ARI score (*p* > 0.05) (Table 3).

### 3.4. Microhardness

**Figure 6:**
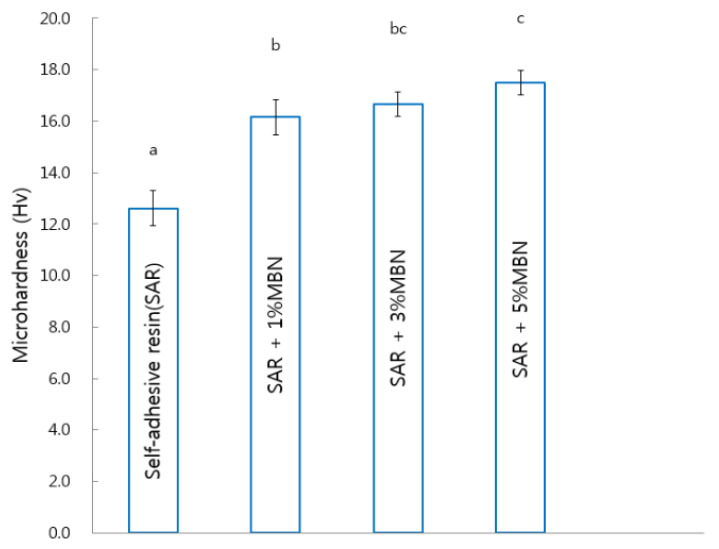
Microhardness comparison between control (SAR) and MBN-incorporated SAR. Labels with the same letters indicate no statistically significant difference between the groups. One-way ANOVA was performed (*n* = 20).

Statistically, the microhardness was found to be greater in SAR + 1% MBN (16.15 ± 0.7 Hv), SAR + 3% MBN (16.66 ± 0.48 Hv), and SAR + 5% MBN (17.50 ± 0.47 Hv) compared to the control group (SAR). As the amount of MBN increased, the microhardness increased (Figure 6). 

### 3.5. Anti-Bacterial Test

For *S. mutans*, comparing the difference between day 1 and day 3, the control group (SAR) showed an increase in value, but the SAR + 1%, 3%, and 5% MBN groups showed statistically reduced values. However, there was no significant difference between the groups.

**Figure 7:**
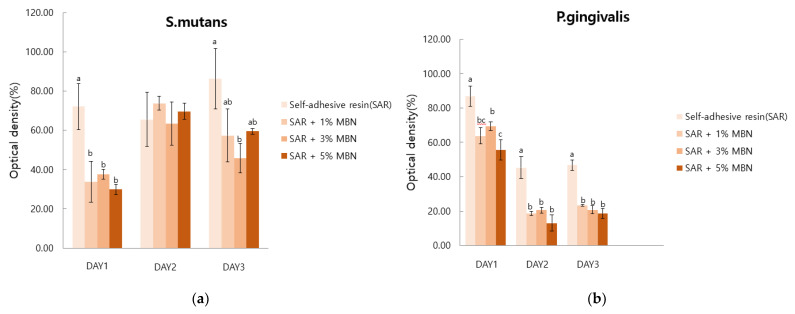
Anti-bacterial effect test of the control (SAR) and MBN-incorporated SAR groups. Labels with the same letters indicate no statistically significant difference between the groups. One-way ANOVA was performed (*n* = 3). (**a**) *S. mutans*, (**b**) *P. gingivalis*.

For *P. gingivalis*, when comparing days 1, 2, and 3, days 2 and 3 showed statistically reduced values for the 1%, 3%, and 5% MBN groups. However, there as no significant difference between the groups (Figure 7).

### 3.6. Degree of Conversion

**Figure 8:**
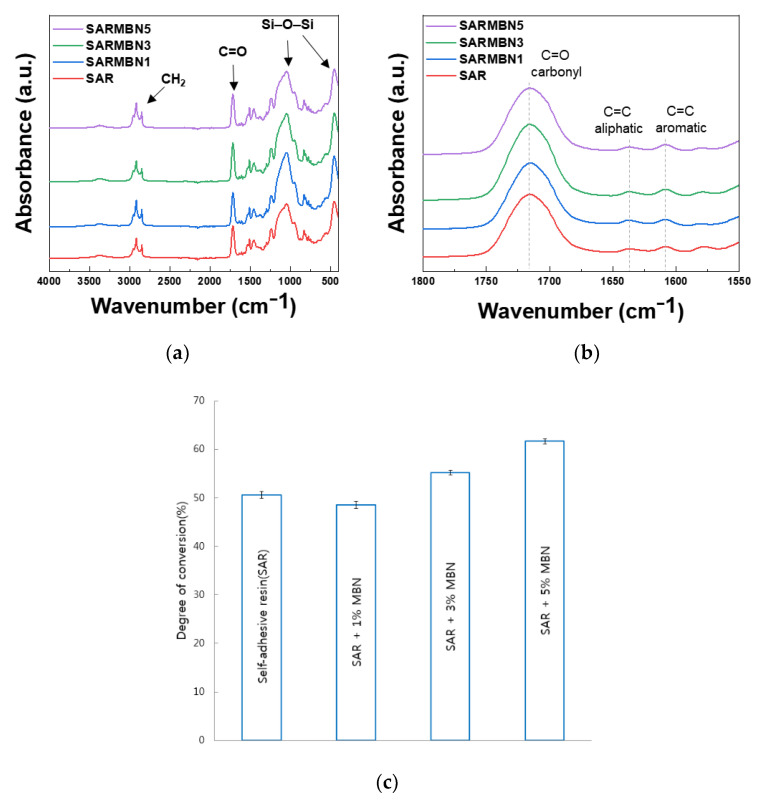
FTIR spectra of SAR and SAR + 1, 3, and 5% MBN; (**a**) 4000–400 cm^−1^; (**b**) 1800–1550 cm^−1^; and (**c**) degree of conversion value of the control (SAR) and MBN-incorporated SAR groups (*n* = 5).

To determine the polymerization and changes of the chemical state of discs, FTIR spectra are shown in Figure 8a,b. The Si-O-Si peaks related to silica were observed at 472 cm^−1^ and 1078 cm^−1^ [16,22,23]. The vibration peaks around 2900 were attributed to the CH_2_, and C=O peaks related to methacrylate were observed around 1717 cm^−1^ [24]. There was no significant difference in the degree of conversion between the control (SAR, 50.58%), SAR + 1% MBN (48.58%), SAR + 3% MBN (55.19%), and SAR + 5% MBN (61.67%) groups (Figure 8c). 

### 3.7. Anti-Demineralization Test

**Figure 9:**
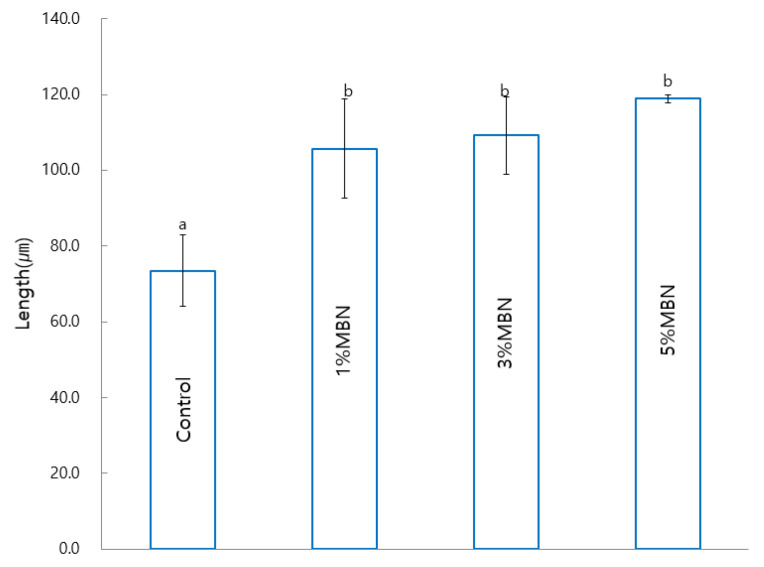
Anti-demineralization test result on the control (SAR) and MBN-incorporated SAR groups. Labels with the same letters indicate no statistically significant difference between the groups. One-way ANOVA was performed (*n* = 5).

Compared to the control group (SAR, 73.5 ± 9.3 μm), the SAR + 1, 3, and 5% MBN groups (105.7 ± 13.0, 109.2 ± 10.2, and 118.8 ± 1.1 μm) showed a significant difference. The remineralization length increased as the MBN content of the sample increased (Figure 9). 

## 4. Discussion

In orthodontic treatment, adhesion of orthodontic attachments is an essential process, but it is also a temporal procedure as the attachments must be removed at the end of treatment. Therefore, the SBS should be sufficiently strong to avoid detachment and sufficiently weak to not damage the enamel layer when detached. In addition, after debonding, the enamel layer should remain intact with the least amount of residue adhesive. To develop self-adhesive materials for functional orthodontic treatment, we added the mesoporous bioactive glass nanoparticle (MBN) to self-etch resin (SAR). The MBN was synthesized using the sol-gel method and evaluated the chemical and structural properties by FTIR, XRD, and TEM. 

The experimental result in this study shows that SAR with MBN (1%, 3%, and 5%) shows statistically higher SBS values than the control (SAR) group. SBS increased in the case of 1% and 3% MBN addition but decreases for the 5% MBN group. This deterioration is affected by the agglomeration of MBN particles. Previous studies also reported a deterioration in the mechanical properties when the MBN mass fraction was 5% [18,25]. However, there was no significant statistical difference between the ARI values between all groups. The ARI score was 3 for all three groups of resin: commercial control adhesive (Transbond XT), control SAR, and SAR with MBN, which indicates that there were no differences in the amount of remnant adhesive. These results conflicted with the study of Shapinko et al. [25], who stated that SAR left a lesser amount of resin residue on enamel. Therefore, we suspect that the added MBN might have resulted in the difference between the properties obtained from the addition or reduction of MBN.

In the microhardness test, there is a statistical difference between the control (SAR) and SAR with MBN (1%, 3%, and 5%) groups. The 1% MBN group shows the lowest value, which indicates the tendency of increasing in accordance with the percentage of MBN content. There is a definite statistical difference between the 1% MBN and 5% MBN groups. According to a study by Khvostenko et. al. [26], in the case of MBN, BAG containing composite resin had a higher filler content, with the microstructure formed as a result of enhanced crack deflection and bridge-forming mechanism, resulting in excellent mechanical properties. Additionally, the chemical binding mechanism between BAG and resin can provide favorable mechanical properties to BAG-containing composite resin. Because the calcium ions released from the BAG react with the carboxylate groups in the resin matrix and the methacrylate groups of the resin is covalently bonded to the Si−OH groups of the BAG [27]. 

There is no statistical difference in the degree of conversion (DC) value, which represents the rate of polymerization between the test groups. According to previous studies, BAG negatively affected the DC value of the experimental resin, which largely depended on the resin system. The DC was the lowest in the bisphenol A diglycidyl methacrylate ethoxylated (Bis-EMA) resin system, followed by the Bis-GMA resin system. In contrast, the DC value of the urethane dimethacrylate (UDMA) resin system did not show any change with the addition of BAG [28]. The resin used in this study, Ortho Connect Flow (GC Corp, Tokyo, Japan) contains 36% urethane dimethacrylate and 34% bisphenol A ethoxylate dimethacrylate, which might explain the lesser change in DC value. It has been hypothesized that the antibacterial properties of bioactive glass cause an increase in the local pH following the exchange of sodium ions with protons in body fluids [29]. Alternating to a highly alkaline environment stresses bacteria, inducing them to modify their form and ultrastructure, thus changing numerous genes and protein phenotype patterns. According to Zhang et al. [30], the destruction of sodium ions elevated the pH to 11 within 8 h and helped maintain a high pH level up to 48 h. Anti-bacterial activity decreased when the media was neutralized, which suggests that this is the principal mechanism of the antibacterial effect of BAG [29]. Other factors contributing to antibacterial activity are the emission of ions, such as silica, calcium, and phosphate, which interfere with bacterial membrane perturbation, resulting in higher osmotic pressure. Therefore, in this study, the addition of MBN to SAR is assumed to enhance the antibacterial effect by affecting both gram-positive and gram-negative bacteria. The results of this study support the above statement by showing a significant antibacterial effect on *S. mutans* and *P. gingivalis*. Although there is no significant difference in the level of antibacterial effectiveness between the different percentage additions of MBN, the result corresponds to the result from previous experiments as the BAG inhibits *S. mutans* and *P. gingivalis* activities [31,32].

Bioactive glass (BAG) is known to form calcium phosphate precipitates in the intraoral area. Owing to this quality, it is well modified in clinical dental materials and considered as a breakthrough in remineralization technology. The current standard treatment of remineralization and prevention of caries of teeth mainly depends on the slow reaction of calcium and phosphorus in saliva [33]. According to Hassanein and El-Brolossy et al. [34], BAG emits highly concentrated calcium, elevating the calcium content around the material and accelerating mineralization. In addition, calcium from BAG increases the active products of apatite ions, leading to the occurrence of apatite nucleation. For the MBN-addition test groups, there is a significant increase in anti-demineralization performance compared to that of the control (SAR) group. Therefore, it is verified that SAR with MBN can increase the occurrence of mineralization, proving its potential as a filler component in restorative dental materials. From the previous results, the following dosage (1 wt%, 3 wt%, and 5 wt% MBN), were appropriate to compare mechanical, antibacterial effect and remineralization ability, and were determined in consideration of the content of silica in the resin. It was found that there was no statistical difference in the physical properties, antibacterial properties, and mineralization ability of the 3% MBN group and 5% MBN group [16,35,36,37]. As mentioned earlier, through the decrease in mechanical properties when the MBN mass fraction is 5% [18,26]. This result corresponds with the results of the research performed by Park et al.; the addition of excessive amounts of MBN may degrade the mechanical properties of the resin [16]. Rather, the 3% MBN group has a similar effect to the 5% MBN group in a small dose, suggesting that the optimal MBN concentration is 3%. 

In this study, we verified the increased bonding strength, resin strength, antibacterial effect, and remineralization ability of the SAR via the addition of MBN. This suggests the possibility of simplifying the clinical procedure, thus aiding the dentist, with the beneficial effects of anti-bacterial activity after bonding, while reducing the possible demineralization effect of the tooth after debonding. 

Similar to previous studies, this experiment contains an extra-orally performed lab-level comparison of the mechanical properties and the anti-demineralization effect of resins, not reflecting any clinical results. Based on the results obtained, a further study must be conducted to test the intraoral application of SAR with MBN for comparison of the bonding strength, mechanical properties, antibacterial effect, and mineralization. In addition, it was previously assumed that the remaining adhesive resin was less in the case of the SAR with MBN than the conventional bonding system after debonding, owing to the primer containing the adhesive agent. However, the current results show that there is no difference in the amount of adhesive residue between the commercial control (Transbond XT) and MBN test groups. Therefore, further studies should be conducted to evaluate the relationship between primer application of the bonding system and the remaining resin on the enamel surface after debonding.

## 5. Conclusions

Since the development of SAR, it took time for SAR to be utilized widely in clinics. However, all the old adhesion systems have not yet been fully replaced. In this study, we have verified the improvement in the mechanical and biological properties of SAR with added MBN. We have demonstrated the potential of supplementing the previous demerits of old SARs. The anti-demineralization property of the material reduces the occurrence of dental caries within the dental bracket area, where it is difficult to maintain dental hygiene. In addition, SAR with MBN saves time and contributes to successful bonding by reducing the bonding failure of the attachment.

## Figures and Tables

**Table 1:** Composition of ortho connect flow.

Composition	Content %
Bisphenol A ethoxylate dimethacrylate	34%
Barium monoxide	5%
Diurethane dimethacrylate, mixture of isomers	36%
α-Alumina	2%
Diboron trioxide	2%
2-Propenoic acid	2%
Benzoic acid, 4-(dimethylamino)	1%
Phenol	0.5%
Silane amine	6%
Phosphine oxide	0.5%
Silica	11%

**Table 2:** Adhesive remnant index (ARI) definition.

Score	Definition
1	All adhesive remained on the tooth
2	Over 90% of the adhesive remained on the tooth
3	10–90% of the adhesive remained on the tooth
4	Below 10% of the adhesive remained on the tooth
5	No adhesive remained on the tooth

**Table 3:** Adhesive remnant index (ARI) score.

Groups	No. of Samples	ARI Scores					Statistics Kruskal-Wallis
		1	2	3	4	5	
Commercial control (Transbond XT)	20	0	0	20	0	0	*p* = 0.567
Self-adhesive resin (SAR)	20	0	0	19	0	1	
SAR + MBN 1%	20	0	0	20	0	0	
SAR + MBN 3%	20	0	0	19	1	0	
SAR + MBN 5%	20	0	0	20	0	0	

## Data Availability

Data sharing is not applicable to this article.
